# Comparison of long-term quality of life based on surgical procedure in patients with rectal cancer

**DOI:** 10.3389/fonc.2023.1197131

**Published:** 2023-05-19

**Authors:** Kotaro Yuge, Keisuke Miwa, Fumihiko Fujita, Kenta Murotani, Takahiro Shigaki, Naohiro Yoshida, Takefumi Yoshida, Kenichi Koushi, Kenji Fujiyoshi, Sachiko Nagasu, Yoshito Akagi

**Affiliations:** ^1^ Department of Surgery, Kurume University School of Medicine, Kurume, Fukuoka, Japan; ^2^ Multidisciplinary Treatment Cancer Center, Kurume University Hospital, Kurume, Fukuoka, Japan; ^3^ Biostatistics Center, Kurume University, Kurume, Fukuoka, Japan

**Keywords:** rectal cancer, surgery, quality of life, long-term, anus-preserving, internal sphincter, low anterior resection, high anterior resection

## Abstract

**Introduction:**

Reports on the long-term quality of life (QOL) over 3 years after surgery in patients who have undergone surgery for rectal cancer are limited. Therefore, we aimed to evaluate the long-term QOL of patients who underwent high anterior resection (HAR), low anterior resection (LAR), internal sphincter resection (ISR), or abdominoperineal resection (APR) for rectal cancer.

**Methods:**

A questionnaire regarding QOL was sent to 360 patients with rectal cancer who underwent curative resection by HAR, LAR, ISR, or APR between January 2005 and December 2015. QOL was assessed using the short-form 36 (SF-36) and modified fecal incontinence QOL (mFIQL) questionnaire. QOL between surgical procedures was analyzed using a multivariate model adjusted for age, sex, and postoperative time.

**Results:**

A total of 144 patients responded with a median follow-up period of 94 months (range 38–233 months). According to surgical procedure, HAR was performed in 26 patients, LAR in 80 patients, ISR in 32 patients, and APR in 6 patients. Patients who underwent HAR had significantly better mFIQL scores than those who underwent LAR and ISR (p=0.013 and p=0004, respectively) and significantly better role/social component summary scores on the SF-36 subscales (p=0.007). No difference was observed in the mFIQL scores between patients who underwent ISR and those who underwent APR (p=0.8423). In addition, postoperative anastomotic leakage sutures did not influence the mFIQL and SF-36 scores after surgery.

**Conclusion:**

The QOL of patients who underwent anus-preserving surgery was best in the HAR group, with the QOL of other groups similar to the APR group. These results suggest that anus- preserving surgery is acceptable from a QOL standpoint. However, a colostomy may be a more satisfactory procedure in some patients.

## 1 Introduction

The problems associated with the surgical treatment of advanced cancer are curability and function preservation. Curative surgical resection is the primary treatment for rectal cancer. Abdominoperineal resection (APR), an anal sphincter non- preserving operation, has been the standard treatment for low rectal cancer since the beginning of the 20th century (1, 2). Anus-preserving surgery has long been controversial owing to oncological and functional reasons. Instrument anastomosis, such as the double-stapler technique developed in the second half of the 20th century, preserves the sphincter muscle. Both low anterior resection (LAR) (3) and internal sphincter resection (ISR) (4, 5) are anus-preserving procedures. These procedures aim to restore the normal process and function of defecation, and improve the patients’ quality of life (QOL) by avoiding permanent colostomy. In addition, ISR has been proposed as a method of preserving the sphincter. As surgery options for lower rectal cancer have been progressing, patient satisfaction has improved as a result of evading permanent colostomy. However, anus-preserving surgery is often associated with evacuative dysfunction and various degrees of incontinence (6–9). Many patients who have undergone ISR experience adverse gastrointestinal effects, such as frequent bowel movements, urgency, and incontinence (6, 10–12), and persistent complaints of postoperative defecation disorders remain. The emphasis on surgery for lower rectal cancer has recently changed to function-preserving surgery; however, there have been no reports comparing QOL between LAR, ISR, and APR.

We aimed to assess long-term functional outcomes and risk factors for functional disorders after lower rectal cancer operations, including LAR, ISR, and APR, through a self-administrated patient questionnaire.

## 2 Materials and methods

### 2.1 Patients

In this study, questionnaires were mailed to 360 patients with rectal cancer who underwent curative resection for the first time at Kurume University Hospital between January 2000 and December 2015. No patients had distant organ metastases. The procedures included high anterior resection (HAR), LAR, ISR, and APR. None of the patients underwent preoperative chemotherapy or radiotherapy. Pre-treatment assessments included digital examination, colonoscopy, computed tomography, and magnetic resonance imaging for staging. Tumor location from the anal verge was assessed using digital examination, colonoscopy, and lower gastrointestinal imaging.

Clinical records and pathological reports were reviewed retrospectively. Informed consent was obtained from each patient, and the study was approved by the Institutional Review Board of Kurume University (approval number: 14152). Clinicopathological findings were diagnosed based on the TNM classification (Seventh edition, 2010).

### 2.2 Surgery

**Figure 1:**
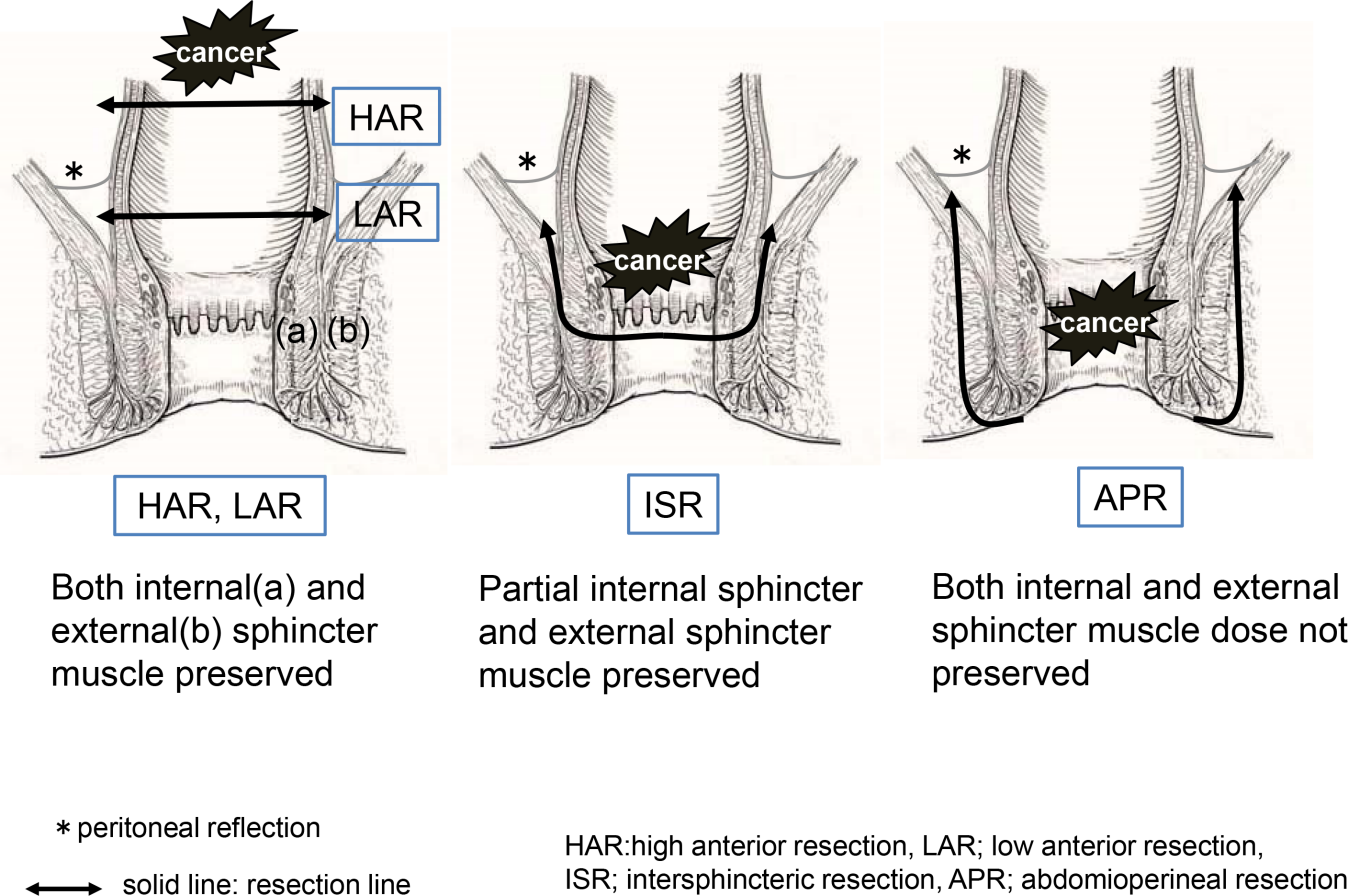
Resection line of the rectum in each surgical procedure.

The technique was standardized in patients undergoing LAR with colorectal anastomosis to include total mesorectal excision and double-stapling anastomosis. A temporary loop ileostomy was performed in all patients who underwent LAR and ISR. The surgical technique for ISR included both abdominal and transanal approaches. In the abdominal approach, total mesorectal excision and pelvic lateral node dissection were performed with or without autonomic nerve preservation. The rectum was mobilized to the pelvic floor as low as possible to facilitate the transanal approach. The anal canal was then circumferentially divided from the puborectal muscle and external sphincter. Most patients underwent end-to-end coloanal anastomosis. Our ISR procedure was performed entirely using hand-sewn anastomosis. Finally, a diverting ileostoma was established and closed 3 months postoperatively or after completion of adjuvant chemotherapy. APR involves dissecting the anal elevator muscle from within the pelvis and the outer fat tissue of the external anal sphincter from the anorectal side. The resection lines for each procedure are illustrated in **Figure 1**.

### 2.3 QOL assessment and questionnaires

Patients were eligible for this study if they had a minimum of 3 years of follow-up and were free of recurrent disease. The exclusion criteria were death, definitive stoma due to anastomotic trouble or poor function, and psychiatric disorders.

The Japanese version of the short form 36 questionnaire (SF-36) was used for a nonspecific general evaluation of QOL (13, 14). The SF-36 comprises eight multi-item scales: physical function, role limitation-physical, bodily pain, general health, vitality, social function, role limitation-emotional, and mental health. Based on these subscales, compartment summary scores can be calculated to provide global measures of physical function (physical component summary, PCS), mental function (mental component summary, MCS), and role/social component summary (RCS). Scale scores range from 0 to 100, with higher scores indicating a better health status.

The Japanese version of the modified fecal incontinence quality of life (mFIQL) questionnaire was used as a specific and sensitive QOL questionnaire (15). The questionnaire explored 14 items, with each response to a specific item assigned a value from 1 to 4 and summarized in a score. The scale scores range from 0 to 100, with higher scores indicating poor QOL. Moreover, since fecal incontinence cannot be assessed in patients that have undergone APR with the mFIQL questionnaire, in this study the questionnaire was set up so that responses would replace “fecal incontinence” with “leakage from ostomy appliance”.

### 2.4 Statistical analysis

The association between each procedure (HAR, LAR, ISR, and APR) and QOL scores (mFIQL, PCS, MCS, and RCS) was analyzed using a multiple linear regression model. The results were analyzed using a multivariate model adjusted for age, sex, and postoperative time. All statistical analyses were performed using SAS version 9.4 (SAS Institute, Inc., Cary, NC, USA), and a p-value less than 0.05 was considered statistically significant.

## 3 Results

### 3.1 Oncological results

Questionnaires were mailed to 360 patients, and 144 completed the survey (40%). No recurrence was observed in any patient. The median follow-up period was 94 months (range 38–233 months). According to surgical technique, HAR was performed in 26 patients, LAR in 80 patients, ISR in 32 patients, and APR in 6 patients. **Table 1** presents the clinical characteristics of the 144 patients analyzed, including the mean tumor distance from the anal verge, type of reconstruction, and lateral lymph node dissection. Overall, the cancer was classified as stage I in 58 patients (40.3%), stage II in 38 (26.4%), and stage III in 44 (30.6%). The mean distal margin from the anal verge in all patients was 4.0 cm (range 0.5–25). The mean distance for each procedure significantly differed between the groups (p<0.0001). Postoperative anastomotic leakage was observed in 15 patients (10.9%; 15/138), including 1 (3.8%) who underwent HAR, 10 (14.7%) who underwent LAR, and 4 (12.5%) who underwent ISR.

**Table 1:** Characteristics of patients who underwent each surgical procedure for rectal cancer.

Variable	Total (n=144)	HAR (n=26)	LAR (n=80)	ISR (n=32)	APR (n=6)	P value
Age, median, (range)	61 (34-86)	60 (47-85)	61 (34-86)	65 (38-75)	69.5 (55-85)	0.3563
Sex						0.1299
male	88	15	52	20	1	
female	56	11	28	12	5	
Pathological stage (TMN)						0.4854
0	5	0	4	0	1	
I	57	7	35	14	1	
II	38	7	20	9	2	
IIIA	33	9	16	7	1	
IIIB	11	3	5	2	1	
Distal margin from AV, mean, cm, (range)	4.0 (0.5-25)	17.0 (5.0-25.0)	4.8 (1.5-12.0)	1.8 (1.0-5.0)	1.5	<.0001
Sphincter preservation		Complete	Complete	Partial	No	
Anastomotic method		Autonomic	Autonomic	Handsewn	–	
Lateral lymph node dissection	5	0	4	1	0	
Anastomotic leakage	15	1	10	4	–	

AV, anal verge.

### 3.2 QOL results

A total of 144 patients who responded to the questionnaire were included in the SF-36 and mFIQL analyses. The changes in the physical, mental, and role/social subscales (PCS, MCS, and RCS) in the SF-36 scores according to the surgical procedure are presented in **Table 2**. No differences in PCS and MCS in SF-36 scores were observed between the procedures (p=0.3256 and p=0.6110, respectively). However, a significant difference was observed in the RCS and mFIQL scores between each procedure (p=0.0057 and p<0.0001, respectively).

**Table 2:** SF-36 and mFIQL scores for each surgical procedure.

QOL	Total (n=144)	HAR (n=26)	LAR (n=80)	ISR (n=32)	APR (n=6)	P value
**SF-36** PCS, median (range)	49.6 (7.4-65.8)	49.8 (16.9-60.1)	50.8 (7.4-62.3)	49.7 (8.7-65.8)	35.5 (25.4-56.6)	0.3256
MCS, median (range)	55.9 (20.5-77.4)	57.0 (39.3-73.8)	55.4 (20.5-77.4)	56.2 (35.6-70.4)	54.4 (42.7-63.2)	0.6110
RCS, median (range)	48.4 (4.7-64.9)	53.2 (4.8-61.8)	48.4 (4.7-64.9)	39.5 (8.8-63.7)	46.3 (14.1-61.6)	0.0057
**mFIQL**, median, (range)	18.4 (0-92.8)	5.3 (0-90.4)	19.5 (0-88.1)	31.1 (0-92.8)	29.1 (14.2-50)	<.0001

**Figure 2:**
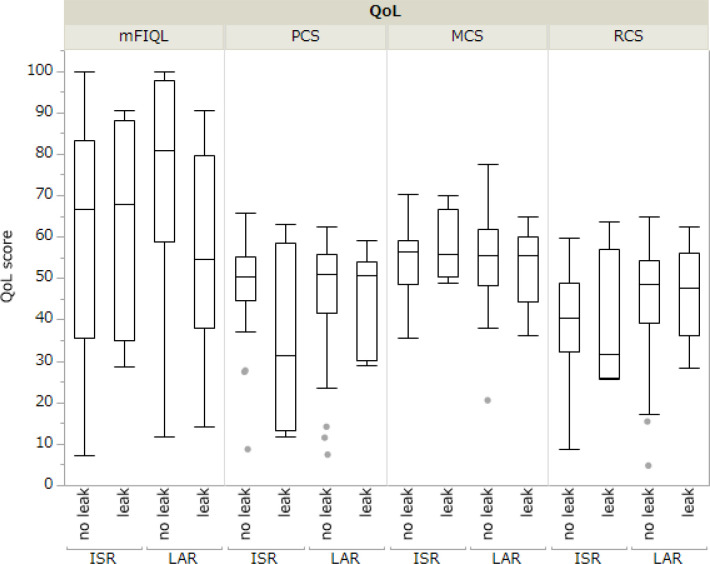
Comparison of QOL in patients with and without postoperative anastomotic leakage (leak).

The comparison between the mFIQL, PCS, MCS, and RCS scores for each surgical procedure is presented in **Table 3**. In the unadjusted model, HAR had a significantly better mFIQL than the other techniques and was significantly better than ISR in the RCS of SF-36. In a model adjusted for age, sex, and time since surgery, HAR was significantly better than LAR and ISR in terms of mFIQL (p=0.013 and p=0.0004, respectively). HAR was also significantly better in the RCS group than in the ISR group (p=0.007). However, no significant differences were observed in the QOL scores between LAR and ISR, LAR and APR, or ISR and APR. In addition, analysis of whether postoperative anastomotic leakage affected QOL revealed that in LAR and ISR, where anastomotic leakage was more common, the presence of anastomotic leakage did not affect long-term QOL (**Figure 2**).

**Table 3:** Comparison between mFIQL, PCS, MCS, and RCS for each surgical procedure.

			mFIQL				PCS				MCS				RCS			
model	Procedure	Reference	Coefficient	95% CI		P value	Coefficient	95% CI		P value	Coefficient	95%CI		P value	Coefficient	95% CI		P value
unadjusted	LAR	HAR	-13.473	-24.87	-2.071	0.0209	1.646	-4.007	7.299	0.5657	-2.621	-6.747	1.504	0.2111	-4.179	-9.797	1.439	0.1436
	ISR		-23.89	-37.22	-10.55	0.0005	1.029	-5.583	7.64	0.7589	-3.14	-7.965	1.685	0.2003	-9.447	-16.018	-2.877	0.0051
	APR		-23.443	-46.31	-0.567	0.0447	-6.93	-18.27	4.411	0.2291	-3.097	-11.373	5.179	0.4607	-6.654	-17.925	4.617	0.2451
	LAR	ISR	10.42	-0.15	20.981	0.0533	0.618	-4.62	5.855	0.816	0.518	-3.304	4.341	0.7889	5.268	0.063	10.473	0.0473
	APR		0.446	-22.02	22.917	0.9687	-7.959	-19.1	3.181	0.16	0.043	-8.086	8.173	0.9916	2.793	-8.278	13.864	0.6187
	LAR	APR	9.97	-11.40	31.35	0.3581	8.576	-2.023	19.176	0.112	0.475	-7.26	8.21	0.9035	2.475	-8.059	13.009	0.643
adjusted*	LAR	HAR	-14.583	-26.04	-3.123	0.013	-0.845	-5.624	3.934	0.7272	-1.719	-5.638	2.2	0.3873	-4.562	-10.13	1.006	0.1075
	ISR		-24.9	-38.38	-11.41	0.0004	-1.999	-7.624	3.625	0.4833	-2.033	-6.645	2.579	0.385	-9.078	-15.63	-2.525	0.007
	APR		-22.591	-45.83	0.651	0.0567	-4.77	-14.46	4.923	0.3322	-3.04	-10.988	4.908	0.4508	-4.163	-15.455	7.129	0.4672
	LAR	ISR	10.317	-0.295	20.929	0.0566	1.154	-3.271	5.58	0.6068	0.314	-3.315	3.923	0.8644	4.515	-0.64	9.671	0.0856
	APR		2.309	-20.58	25.205	0.8423	-2.771	-12.32	6.778	0.567	-1.007	-8.837	6.823	0.7996	4.914	-6.21	16.039	0.3839
	LAR	APR	8.008	-13.95	29.967	0.472	3.925	-5.232	13.083	0.3981	1.321	-6.188	8.83	0.7285	-0.399	-11.068	10.269	0.9412

* adjusted for age, sex, and postoperative time.

## 4 Discussion

The standard treatment for curable rectal cancer is surgical resection, but in the lower rectum, this has a significant impact on postoperative QOL. Thus, we must consider the individual disease condition and the QOL of patients with rectal cancer. We aimed to examine the postoperative QOL of patients who underwent surgery for rectal cancer. There have been few reports on the long-term QOL after surgery in patients who have undergone surgery for rectal cancer. This study reports the long-term QOL of patients who underwent individual rectal cancer surgeries.

Regarding QOL after LAR versus HAR, Vironen et al. reported no significant difference in QOL between LAR and HAR (16). However, this may be because the QOL Questionnaire for Colorectal Cancer Patients, which includes a QOL questionnaire and a scale regarding defecation problems, was not used. In contrast, there have been several reports of better QOL after HAR than after LAR (17, 18). In our study, HAR was generally better than the other procedures in terms of the mFIQL and RCS of SF-36, supporting previous reports.

Many patients with rectal cancer require anus-preservation during surgery. Therefore, it is necessary to determine the long-term QOL outcomes in patients who undergo anus-preserving surgery. Anus- preserving surgery with LAR or ISR was approved based on oncological outcomes in patients with lower rectal cancer (3, 5). Therefore, we evaluated the QOL, especially between APR and anus-preserving surgery, in patients with lower rectal cancer. In one study, LAR resulted in a lower QOL than APR, albeit in the short term (17). However, in another study, LAR resulted in a better QOL than APR (18). In this study, no significant difference was observed between LAR and APR for either SF-36 or mFIQL. A recent report indicated that postoperative QOL declined the most at 6 and 12 months; at 24 months, however, no difference was observed in the QOL with or without sphincter preservation (19). The results of our study support this report because of the long observation period; however, they are still controversial.

The advance of surgical procedures and tools and a deeper understanding of the conditions for obtaining a safe distal resection margin, which can be as short as 1–2 cm, have allowed the increased use of sphincter-saving procedures without compromising oncological outcomes (20, 21). ISR is an alternative to APR when the tumor is in the lower rectum (22). However, no reports have evaluated long-term QOL following ISR, and this is the first report of its kind. In this study, we observed no significant difference in SF-36 and mFIQL between the 32 patients who underwent ISR and the 6 patients who underwent APR; we consider these valuable data on long-term QOL (**Supplementary Tables S1** and **S2**). QOL scores for APR did not improve over time, and reports indicate that patients with stoma have significantly worse QOL scores than those without (18). We had previously agreed with this report; however, in this study, we observed no significant difference between ISR and APR. Recently, it has been reported that patients with low anastomoses had a lower global QOL at 24 months compared to patients with permanent stomas (19). These continence function disorders can have such a strong impact on QOL that colostomy might be a more satisfactory operation for some patients (17).

Sphincter-sparing procedures are now performed more frequently, which is a positive development in this field. However, the effect of sphincter preservation on the patient’s QOL should not be underestimated, and the possibility that preservation of the anus may reduce QOL more than APR needs to be re-examined. The possibility that the QOL after ISR may be inferior to that after APR must also be fully explained to patients before surgery, and the surgeon must be aware of this. At the same time, the patients’ psychological aspects must also be considered. All patients who underwent ISR underwent temporary stoma management. Their long-term QOL was impaired due to defecation problems; however, none of them requested revision surgery to create a permanent stoma. This indicates the need for a more in-depth investigation of the psychological burden of patients with stomas.

Some reports have revealed that postoperative anastomotic leakages after rectal cancer worsens prognosis (23). We considered that postoperative anastomotic leakage partially influenced QOL. In this study, all patients in the ISR and LAR groups who developed postoperative anastomotic leakage improved conservatively with appropriate drainage and antimicrobial therapy, and no patients underwent salvage surgery. Regarding the presence or absence of anastomotic leakage, there were no significant differences in QOL assessments, such as the SF-36 (PCS/MCS/RCS) and mFIQL. In addition, no significant differences in QOL assessments were found between LAR and ISR when narrowed down to cases with anastomotic leakage (**Supplementary Figure S1**). These results indicate that anastomotic leakage has no long-term influence on patient QOL after rectal cancer surgery. However, we believe that the relationship between postoperative anastomotic leakage and mFIQL should continue to be a focus of attention, as the observed lack of statistical difference may be due to sample size.

Our study had several limitations that should be acknowledged. First, the response rate to the questionnaire was low, resulting in a small sample size. We consider that this low response rate was largely due to the long postoperative period and the fact that many patients had already completed follow-up. Second, the QOL for each procedure was not evaluated during the same postoperative period. Third, the patients’ lifestyle, including their work, was not considered. Fourth, objective measurements of anorectal function obtained alongside QOL evaluation were lacking. And finally, in this study we defined “long-term QOL” as QOL at more than 3 years after surgery. In the future, it is desirable to prospectively investigate true “long-term QOL” by repeatedly evaluating QOL over a long period of time, including lifestyle and anal function, postoperatively for each surgical procedure. Further, QOL, including psychological aspects, should be investigated in patients who have undergone temporary stoma management. The goal is to determine which patients would benefit the most from each type of surgery, considering their life circumstances.

In conclusion, based on our study, the QOL of patients who underwent anus-preserving surgery was the best in the HAR group, with the QOL of other groups similar to the APR group. These results suggest that anus-preservation surgery is acceptable from oncological and QOL standpoints. However, a colostomy may be a more satisfactory procedure in some patients. Based on our results, clinicians should consider all influencing factors, including psychological factors, for QOL in patients with rectal cancer.

## Data availability statement

The raw data supporting the conclusions of this article will be made available by the authors, without undue reservation.

## Ethics statement

The studies involving human participants were reviewed and approved by Institutional Review Board of Kurume University (approval number: 14152). The patients/participants provided their written informed consent to participate in this study.

## Author contributions

KY conceived the study, conducted the questionnaire survey, collected the data, and wrote the manuscript. FF and YA contributed substantially to the conception and design of this study. KMu and KMi were involved in the statistical analysis of the raw data and revised the manuscript. TS, NY, TY, KK, KF, and SN collected the clinical data and contributed to data entry, management, and protection. YA contributed greatly to the creation of figures, and SN contributed to the creation of tables. KMi reviewed and edited the manuscript. All authors discussed the results and contributed to the final manuscript. All authors contributed to the article.
