# Supplementary material for: Comparison of long-term quality of life based on surgical procedure in patients with rectal cancer
Source: Front Oncol. 2023 May 19;13:1197131. doi: 10.3389/fonc.2023.1197131 (PMC10235785; doi:10.3389/fonc.2023.1197131)
Supplement: Supplementary file 1 [file Table_1.docx]

Table S1. QOL scores for 32 individuals who underwent ISR

|  | **Age** | **Sex** | **PCS** | **MCS** | **RCS** | **mFIQL** | **Anastomotic leakage** | **Postoperative**  **time (month)** |
| --- | --- | --- | --- | --- | --- | --- | --- | --- |
| **1** | 49 | male | 62.4 | 56.2 | 45.6 | 33.3 | - | 156.6 |
| **2** | 54 | male | 51.4 | 56.4 | 57.7 | 0.0 | - | 147.0 |
| **3** | 66 | female | 27.4 | 50.4 | 32.0 | 78.6 | - | 140.7 |
| **4** | 72 | female | 27.7 | 48.5 | 34.7 | 11.9 | - | 134.7 |
| **5** | 66 | male | 45.4 | 55.2 | 36.6 | 29.0 | + | 119.7 |
| **6** | 65 | female | 37.1 | 59.0 | 40.5 | 4.8 | - | 119.7 |
| **7** | 56 | male | 51.7 | 64.4 | 48.6 | 23.8 | - | 111.6 |
| **8** | 74 | female | 51.7 | 56.4 | 53.5 | 4.8 | - | 123.3 |
| **9** | 56 | male | 50.8 | 46.8 | 59.6 | 23.8 | - | 122.8 |
| **10** | 51 | male | 59.2 | 47.5 | 45.7 | 69.0 | - | 110.4 |
| **11** | 71 | male | 8.7 | 51.0 | 8.8 | 92.9 | - | 92.9 |
| **12** | 74 | male | 47.1 | 61.6 | 48.9 | 25.0 | - | 92.4 |
| **13** | 58 | male | 11.7 | 70.0 | 63.7 | 19.0 | + | 91.5 |
| **14** | 65 | male | 45.5 | 57.9 | 54.7 | 16.7 | - | 91.9 |
| **15** | 69 | male | 45.6 | 59.7 | 47.6 | 52.4 | - | 102.0 |
| **16** | 60 | male | 44.6 | 39.8 | 35.9 | 76.2 | - | 96.9 |
| **17** | 38 | female | 63.1 | 56.4 | 26.9 | 0.0 | + | 84.0 |
| **18** | 65 | female | 52.3 | 41.0 | 34.5 | 58.1 | - | 82.9 |
| **19** | 75 | female | 17.3 | 48.9 | 25.7 | 69.0 | + | 93.8 |
| **20** | 47 | male | 56.5 | 55.7 | 38.6 | 47.6 | - | 80.5 |
| **21** | 69 | male | 49.3 | 55.6 | 58.6 | 0.0 | - | 79.1 |
| **22** | 59 | male | 56.4 | 60.4 | 33.9 | 33.3 | - | 72.4 |
| **23** | 54 | female | 55.1 | 57.1 | 40.6 | 19.0 | - | 72.1 |
| **24** | 41 | male | 55.0 | 40.5 | 32.6 | 52.4 | - | 67.2 |
| **25** | 56 | female | 41.6 | 49.0 | 31.2 | 43.1 | - | 80.9 |
| **26** | 74 | female | 47.0 | 57.0 | 57.0 | 9.5 | - | 61.6 |
| **27** | 54 | male | 65.8 | 35.6 | 27.7 | 33.3 | - | 71.4 |
| **28** | 75 | male | 43.9 | 60.0 | 31.5 | 47.6 | - | 64.9 |
| **29** | 62 | male | 55.3 | 59.1 | 40.7 | 16.7 | - | 64.5 |
| **30** | 71 | male | 58.6 | 70.4 | 46.7 | 16.7 | - | 51.8 |
| **31** | 72 | female | 50.2 | 57.7 | 19.4 | 63.8 | - | 44.1 |
| **32** | 69 | female | 45.8 | 52.6 | 32.3 | 64.3 | - | 38.8 |
| **Mean±SD** | 62.1±10.1 |  | 46.2±14.0 | 54.3±8.1 | 40.3±12.6 | 35.5±26.1 |  | 92.6±29.6 |
