# Supplementary material for: Comparison of long-term quality of life based on surgical procedure in patients with rectal cancer
Source: Front Oncol. 2023 May 19;13:1197131. doi: 10.3389/fonc.2023.1197131 (PMC10235785; doi:10.3389/fonc.2023.1197131)
Supplement: Supplementary file 2 [file Table_2.docx]

|  | **Age** | **Sex** | **PCS** | **MCS** | **RCS** | **mFIQL** | **Anastomotic**  **leakage** | **Postoperative**  **time (month)** |
| --- | --- | --- | --- | --- | --- | --- | --- | --- |
| **1** | 55 | female | 56.6 | 49.1 | 37.2 | 19.0 | - | 129.9 |
| **2** | 60 | female | 44.4 | 63.3 | 52.9 | 14.3 | - | 104.1 |
| **3** | 68 | male | 34.2 | 42.8 | 39.8 | 50.0 | - | 97.8 |
| **4** | 72 | female | 25.4 | 62.9 | 61.7 | 21.4 | - | 62.0 |
| **5** | 85 | female | 32.4 | 59.7 | 53.3 | 36.9 | - | 63.3 |
| **6** | 71 | female | 37.0 | 48.4 | 14.1 | 45.2 | - | 55.1 |
| **Mean±SD** | 68.5±10.4 |  | 38.2±10.9 | 54.3±8.7 | 43.2±16.9 | 31.2±14.9 |  | 85.3±29.8 |

Table S2. QOL scores for 6 individuals who underwent APR
