# Supplementary material for: Comparison of long-term quality of life based on surgical procedure in patients with rectal cancer
Source: Front Oncol. 2023 May 19;13:1197131. doi: 10.3389/fonc.2023.1197131 (PMC10235785; doi:10.3389/fonc.2023.1197131)
Supplement: Supplementary file 3 [file Image_1.pdf]

Figure S1 Comparison of QOL in patients with postoperative anastomotic leakage

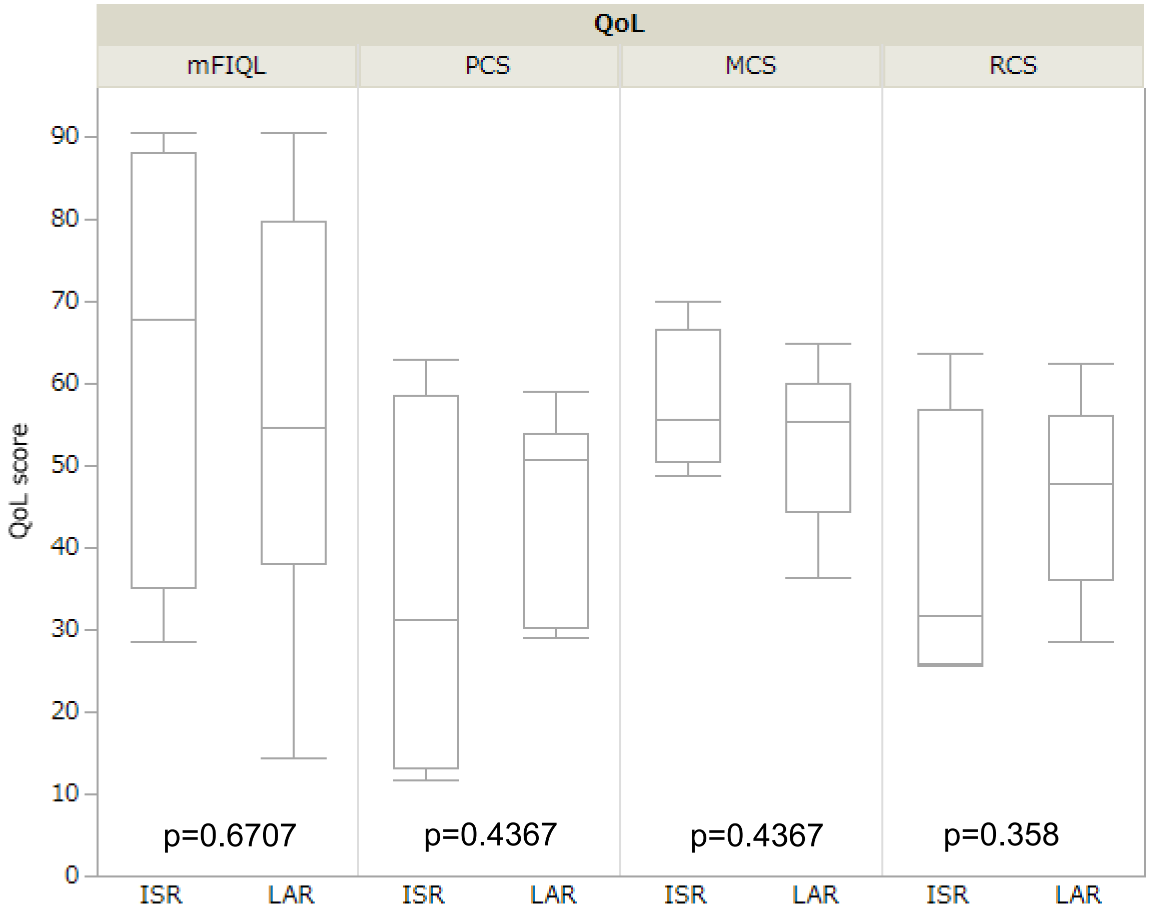

Statistical analysis was performed with the Mann-Whitney U test.
